# Setup of an Ultrasonic-Assisted Extraction to Obtain High Phenolic Recovery in *Crataegus monogyna* Leaves

**DOI:** 10.3390/molecules26154536

**Published:** 2021-07-27

**Authors:** Beatriz Martín-García, María del Carmen Razola-Díaz, Ana María Gómez-Caravaca, Guillermo Benítez, Vito Verardo

**Affiliations:** 1 Department of Analytical Chemistry, Faculty of Sciences, University of Granada, Avd. Fuentenueva s/n, 18071 Granada, Spain; bea91mg@ugr.es (B.M.-G.); anagomez@ugr.es (A.M.G.-C.); 2 Department of Nutrition and Food Science, University of Granada, Campus of Cartuja, 18071 Granada, Spain; carmenrazola@correo.ugr.es; 3 Institute of Nutrition and Food Technology ‘José Mataix’, Biomedical Research Center, University of Granada, Avda del Conocimiento s/n., 18100 Granada, Spain; 4 Department of Botany, Campus Universitario de Cartuja, University of Granada, 18071 Granada, Spain; gbcruz@ugr.es

**Keywords:** ultrasonic-assisted extraction, Hawthorn (*Crataegus monogyna*), HPLC-ESI-TOF-MS, HPLC-FLD, Box–Behnken design

## Abstract

Hawthorn leaves are a rich source of phenolic compounds that possess beneficial activities for human health. Ultrasonic-assisted extraction (UAE) is an extraction technique frequently used for the isolation of phenolic compounds in plants. Thus, in this study, a Box–Behnken design was used to optimize UAE conditions such as the percentage of acetone, the extraction time and solvent-to-solid ratio (*v*/*w*) in order to obtain the maximum content of total compounds by Folin–Ciocalteu and the maximum in vitro antioxidant activity by DPPH, ABTS and FRAP assays in *Crataegus monogyna* leaves. The optimum conditions to obtain the highest total phenolic content and antioxidant activities were 50% acetone, 55 min and 1/1000 (*w*/*v*). A total of 30 phenolic compounds were identified and quantified in *C. monogyna* leaf extract obtained at these optimum UAE conditions. HPLC-MS allows the identification and quantification of 19 phenolic compounds and NP-HPLC-FLD analyses showed the presence of 11 proanthocyanidins. According to the results, the most concentrated phenolic compounds in *C. monogyna* leaf extract obtained at optimum UAE conditions were phenolic acid derivatives such as protocatechuic acid-glucoside, dihydroxy benzoic acid pentoside and chlorogenic acid, flavones such as 2″-*O*-rhamnosyl-*C*-hexosyl-apigenin, flavonols such as hyperoside and isoquercetin and proanthocyanidins such as monomer and dimer. As a result, the optimized UAE conditions could be used to obtain an extract of *C. monogyna* leaves enriched with phenolic compounds.

## 1. Introduction

*Crataegus* species, also known as hawthorn (family: *Rosaceae*), are plants extensively distributed in the northern hemisphere and are used to provide many natural health products such as tablets, teas and aqueous extracts [1,2]. The genus *Crataegus* comprises between 150 and 1200 species depending on the species concept employed [3]. *Crataegus* *monogyna* Jacq. and *Crataegus laevigata* (Poir.) DC. are the major hawthorn species in Middle Europe, *Crataegus granatensis* Boiss., *Crataegus laciniata* Ucria., *Crataegus* *pentagyna* Waldst. and Kit. ex Willd., *Crataegus nigra* Waldst. and Kit., and *Crataegus azarolus* L. in Southern and Southeastern Europe, and *Crataegus pinnatifida* Bunge. and *Crataegus scabrifolia* (Franch.) Rehder. in China [4,5]. *Crataegus* species are a rich source of bioactive compounds such as phenolic compounds, triterpenoids and vitamins [6]. Hawthorn fruits, leaves and flowers contain phenolic compounds such as flavone and flavonol glycosides, hydroxycinnamic acids, flavan-3-ols (especially (−)-epicatechin) and flavan-3-ol oligomers (B-type procyanidins). [7,8,9,10,11]. Flavan-3-ols (epicatechin), flavonols (hyperoside) and hydroxycinnamic acids such as chlorogenic acid and protocatechuic acid are dominant in leaves [11]. These phenolic compounds have shown beneficial effects on human health including neuroprotective, hepatoprotective, cardioprotective, nephroprotective, anti-inflammatory, gastroprotective and antimicrobial activities which are attributed in part to their antioxidant activity [7,12]. The most important natural health products are those directed at treating cardiovascular diseases, which account for over 25% of phytomedicine sales in the European Union [13]. These products include those made from *Crataegus* species (hawthorn), which are widely available in Europe, Asia, and North America. Available products include tinctures, tablets, teas and aqueous extracts of *Crataegus* leaves, flowers and fruits [14]. Indeed, *C. monogyna*, *C. laevigata* and their hybrids are allowed by European Pharmacopoeia for the preparation of phytomedicines [15]. The extraction technique is the most important step in order to obtain a high recovery of phenolic compounds from hawthorn samples. The isolation of phenolic compounds from hawthorn samples depends on some extraction factors including the extraction temperature, extraction time and extraction solvent. Therefore, it seems important to evaluate the impact of the experimental conditions of extraction on the daily intake dose and its reproducibility [16]. Previous studies have used ultrasonic-assisted extraction (UAE) as an efficient technique in the recovery of phenolic compounds in hawthorn leaves, employing ethanol, methanol, water or mixtures of these solvents [2,7,8]. Other mixtures, such as acetone with water, are used to extract high-molecular-weight phenolic compounds such as oligomeric and polymeric flavan-3-ols from hawthorn leaves and flowers (*Crataegi folium cum flore*) from *Crataegus* spp. (*Rosaceae*) [17]. Among the methods used for the determination of hawthorn phenolic compounds, the most used is high-performance liquid chromatography (HPLC), coupled with diode array detection (DAD) and mass spectrometry (MS), enabling the determination of individual phenolics [1]. Nevertheless, oligomers of proanthocyanidins (higher than trimers) are analyzed by normal-phase (NP)-HPLC coupled with a fluorimetric detector (FLD), which allows for their separation according to their degree of polymerization using a silica gel column [18,19].

The aim of the present study was the establishment for the first time of optimized ultrasonic-assisted extraction factors such as acetone/water composition, extraction time and ratio of the plant material to solvent by using a Box–Behnken design to obtain the highest phenolic content from *C. monogyna* leaves and the highest in vitro antioxidant activity measured by DPPH, FRAP and ABTS assays. For that purpose, the determination of phenolic compounds in *C. monogyna* leaves obtained by UAE by using HPLC-MS was carried out. In addition, proanthocyanidins were quantified by NP-HPLC-FLD.

## 2. Results and Discussion

### 2.1. Fitting the Model

The Box–Behnken experimental design elaborated for the optimization of UAE conditions, considering experimental values obtained for the variable responses, is exhibited in Table 1.

The regression coefficients of the models and the results of the analysis of variance (ANOVA) are shown in Table 2. The evaluation of the model was carried out according to the significance of the regression coefficients, quadratic correlation coefficients (R^2^), quadratic correlation coefficients adjusted (R^2^ adjusted), coefficient of variation (CV) and lack of fit. According with previous studies, the level of significance was α < 0.1 in order to increase the number of significant variables [20,21]. The significant variables on the response variable of TPC were the linear effect of acetone/water % (*v*/*v*) (X_1_) (*p* = 0.047803) and its quadratic effect (X_11_) (*p* = 0.040422), linear effect of time (X_2_) (*p* = 0.009215) and its quadratic effect (X_22_) (*p* = 0.009880), the linear effect of solvent-to solid ratio (*v*/*w*) (X_3_) (*p* = 0.043882) and its quadratic effect (X_33_ = 0.005158) and the cross effect between acetone/water % (*v*/*v*) with solvent-to-solid ratio (*v*/*w*) (X_23_). The significant variables on the variable response of DPPH were the linear effect of acetone/water % (*v*/*v*) (X_1_) (*p* = 0.021940) and its quadratic effect (X_11_) (*p* = 0.012593), linear effect of time (X_2_) (*p* = 0.033790) and its quadratic effect (X_22_) (*p* = 0.014293), the linear effect of solvent-to solid ratio (*v*/*w*) (X_3_) (*p* = 0.027678) and its quadratic effect (X_33_ = 0.017333) and the cross effect of acetone/water % (*v*/*v*) with time (X_12_) (*p* = 0.060553). In addition, the significant effects on the response ABTS were the following: acetone/water % (*v*/*v*) (X_1_) (*p* = 0.005654) and its quadratic effect (X_11_) (*p* = 0.004257), linear effect of time (X_2_) (*p* = 0.012604) and its quadratic effect (X_22_) (*p* = 0.029806), the linear effect of solvent-to solid ratio (*v*/*w*) (X_3_) (*p* = 0.008358) and its quadratic effect (X_33_= 0.006386), the cross effect between acetone/water % (*v*/*v*) and time (X_12_) (*p* = 0.033970), the cross effect between acetone/water % (*v*/*v*) and solvent-to-solid ratio (*v*/*w*) (X_13_) (*p* = 0.053264) and time with solvent-to-solid ratio (*v*/*w*) X_23_ (*p* = 0.053382).

An analysis of variance (ANOVA) with a 95% confidence level was generated and the effect and regression coefficients of individual linear, quadratic and interaction terms were determined. The models presented a high correlation between independent factors and response variables with quadratic correlation coefficients (R^2^) from 92.19–97.54%, except for the variable response of TPC and DPPH, whose responses possess a good correlation but lower than the other ones (R^2^ = 80.64 and 85.97%). According to Le Man et al. (2010), a model is adequate when R^2^ > 0.75 [22]. The R^2^ of each response is in close range with the adjusted R^2^ with a variation coefficient between 0.197–0.811%. Additionally, the *p* value of lack-of-fit was used to verify the adequacy of the model, which was non-significant (*p* > 0.05), thus, the model fits well (Table 2). Moreover, models were statistically acceptable since the *p* value was lower than 0.05 for all cases.

### 2.2. Analysis of Response Surfaces

**Figure 1:**
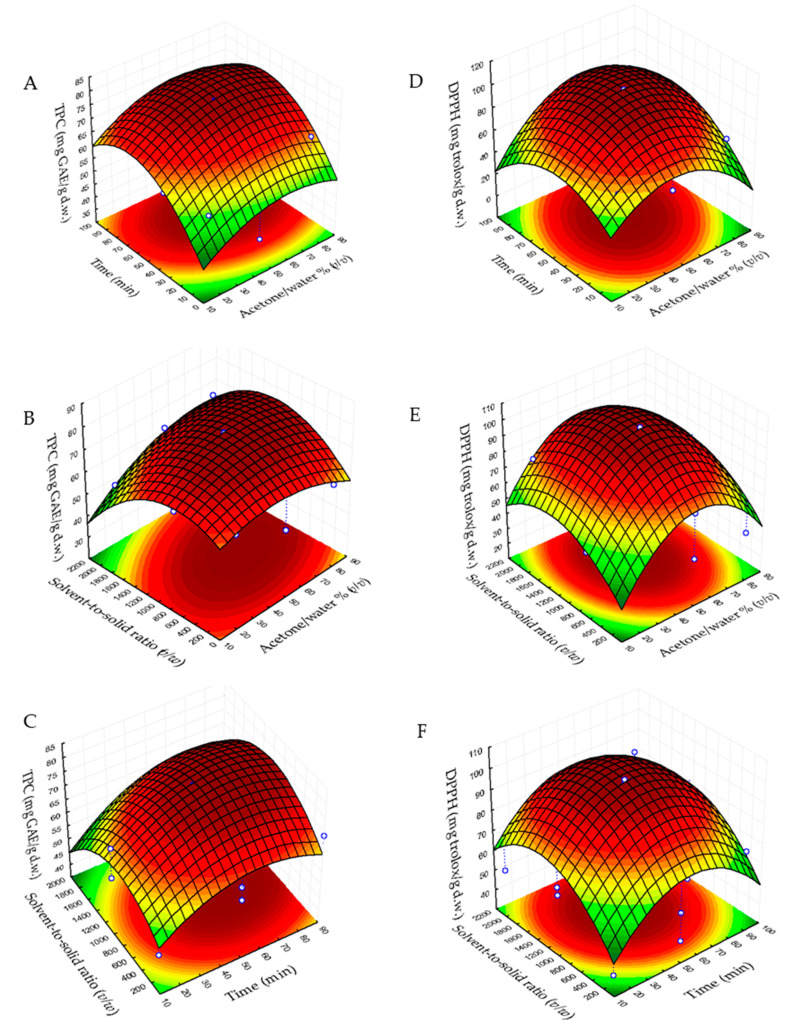
Response surface plots showing combined effects of process variables for total phenolic content and DPPH assay. (**A**,**D**) Solvent-to-solid ratio (*v*/*w*) vs. acetone/water % (*v*/*v*); (**B**,**E**) acetone/water (*v*/*v*) vs. time; and (**C**,**F**) solvent-to-solid ratio (*v*/*w*) vs. time.

**Figure 2:**
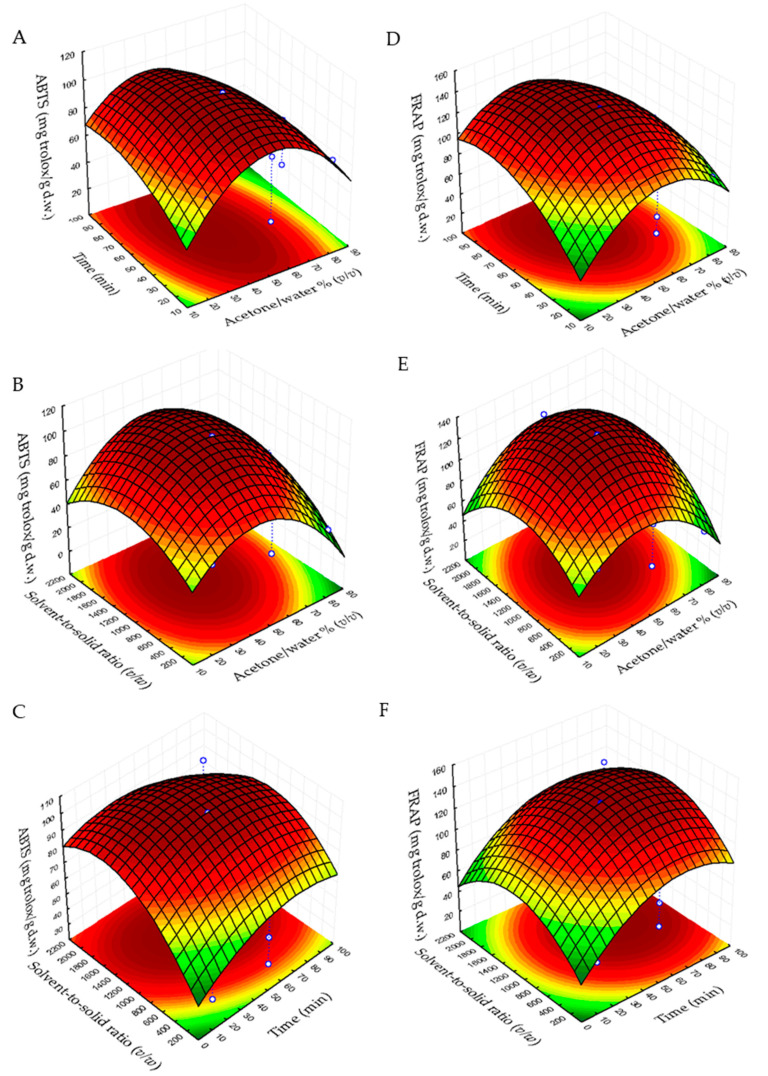
Response surface plots showing combined effects of process variables for ABTS and FRAP assays. (**A**,**D**) Solvent-to-solid ratio (*v*/*w*) vs. acetone/water % (*v*/*v*); (**B**,**E**) acetone/water (*v*/*v*) vs. time; and (**C**,**F**) solvent-to-solid ratio (*v*/*w*) vs. time.

Figure 1 and Figure 2 are the three-dimensional plots showing the effects of acetone/water % (*v*/*v*) (X_1_) with time (X_2_) (A, D), of acetone/water % (*v*/*v*) (X_1_) with solvent-to-solid ratio (*v*/*w*) (X_3_) (B, E) and time (X_2_) with solvent-to-solid ratio (*v*/*w*) (X_3_) (C, F) on the total phenolic content, DPPH, ABTS and FRAP.

Figure 1A shows the maximum TPC in the range of 45–75% acetone/water and 55–75 min, whereas in Figure 1B its maximum concentration is observed at 50–70% acetone/water and 700–1200 solvent-to-solid ratio (*v*/*w*), and in Figure 1C its maximum value shows at 50–70 min and 700–1200 solvent-to-solid ratio (*v*/*w*). Regarding DPPH variable responses, in Figure 1D its maximum content can be seen in a range of 45–65% acetone/water at 45–65 min, in Figure 1E the maximum content of DDPH shows in the range of 1000–1500 solvent-to-solid ratio (*v*/*w*) and 45–65% acetone/water, whereas in Figure 1F its maximum content shows at 1000–1500 solvent-to-solid ratio (*v*/*w*) and 45–70 min. In addition, concerning ABTS, in Figure 2A its maximum value is observed between 40–55% acetone/water at 35–75 min. In Figure 2C the maximum content of ABTS is observed at 35–55 min and 900–1600 solvent-to-solid ratio (*v*/*w*) and in Figure 2C the maximum value of this response can be observed at 1000–1500 solvent-to-solid ratio (*v*/*w*) at 40–70 min. Finally, the maximum content of FRAP can be observed at 35–55% of acetone/water at 50–85 min (Figure 2D), 40–60% acetone/water and 800–500 solvent-to solid ratio (*v*/*w*) (Figure 2E) and 800–1500 solvent-to-solid ratio (*v*/*w*) at 50–85 min (Figure 2F).

### 2.3. Optimization of Ultrasonic-Assisted Extraction

After determination of the optimal conditions through the three-dimensional plots, the final step of the RSM was to predict the accuracy of the mathematical model.

Results of the optimal conditions to obtain the highest total phenolic content and in vitro antioxidant activity by DPPH, ABTS and FRAP from *C. monogyna* are shown in Table 3. Optimal conditions were the same for all responses, which were 50% acetone/water, 55 min and 1000 solvent-to-solid ratio to obtain predictable values of 78 ± 4 mg GAE/g d.w. for total phenolic content, 98 ± 11, 102 ± 8 and 133 ± 13 mg Trolox/g d.w. for DPPH, ABTS and FRAP. Extraction time was lower than reported in a previous study that reported 1.5 h to obtain a maximum yield of flavonoids from hawthorn seed, which was 16.45 ± 0.02 mg/g d.w. obtained at a solvent-to-solid ratio of 18 (*v*/*w*) and 72% ethanol [23]. A previous study reported a similar total phenolic content in *C. pentagyna* (98.3–107 and 128 mg GAE/g d.w.), which was extracted by maceration (0.2 g of leaves with 15 mL of 80% acetone for 48 h) followed by an ultrasound-assisted extraction of 15 min with 10 mL of 80% acetone, five successive times [11]. The total phenolic content obtained in *C. monogyna* leaf extract at optimum conditions was in the magnitude order than a previous study in an extract of *C. orientalis* leaves, which was 94.2 mg GAE/g d.w. Nevertheless, this previous study reported an extraction in a Soxhlet apparatus for 72 h with 96% ethanol [24]. Therefore, it has been shown that ultrasonic-assisted extraction is more efficient than conventional extraction techniques due to requiring lower extraction times to obtain a similar phenolic recovery from *Crataegus* leaves. In addition, Alirezalu et al. [12] reported a similar phenolic content of 76.74 ± 0.80 mg GAE/g d.w. in *C. monogyna* leaf extract obtained by ultrasound (for 30 min at 25 °C) using methanol/water (80%, *v*/*v*). However, this previous study reported a total phenolic content of 33.88 ± 0.28 mg GAE/g d.w. in other *C. monogyna* leaf extract obtained at other locations, which was 56.6% lower than that obtained in the present study [25]. Therefore, data show significant differences in total phenolic content of hawthorn due to several factors such as natural habitat, genotype, growth stage, extraction procedure and method for determination of total phenolics [24].

### 2.4. Identification of Phenolic Compounds C. monogyna Leaf Extract at Optimum UAE conditions by HPLC-MS

*C. monogyna* leaf extract obtained at optimum UAE conditions was analyzed by HPLC coupled to MS with a TOF analyzer. Phenolic compounds were identified by rendering their mass spectra, bearing in mind the data reported in the literature and, when available, by co-elution with commercial standards and using several databases. Parameters which allowed the identification of these phenolic compounds were retention time, *m*/*z* experimental and calculated, error and Fit Conf %, mainly in source fragments and molecular formulae (M-H)^−^.

A total of 19 phenolic compounds were identified in *C. monogyna* leaf extract obtained at optimal conditions (Table 4). Peak 1 at 1.93 min at *m*/*z* 315.0719 reported a molecular formula of C_13_H_15_O_9_ and fragment ions at *m*/*z* 153.0171 and 109.0246, which corresponds to protocatechuic acid-glucoside as previously reported by Żurek et al. 2021 in berries of *Crataegus* [26]. Peak 2 at 2.41 min with *m*/*z* 299.0759 with a molecular formula of C_13_H_15_O_8_ and fragment ions at *m*/*z* 137.0249 was identified as hydroxybenzoylhexose, which has been identified previously in *Rorippa indica* (L.) Hiern [27]. Peak 3 at 3.63 min with a molecular ion at *m*/*z* 285.0606 C_12_H_13_O_8_ and fragment ions at *m*/*z* 152.0104, 153.0187, 108.0197 and 109.0269 was proposed to be dihydroxy-benzoic-acid-pentoside (C_12_H_13_O_8_), which has been identified previously in *Sclerocarya birrea* (A.Rich.) Hochst [28]. Peak 4 at 5.32 min presented a molecular ion at *m*/*z* 515.1407 with a molecular formula of C_22_H_27_O_14_ with fragment ions at *m*/*z* 191.0552, 161.0229 and 323.0756 corresponding to 5-*O*-(3′-*O*-caffeoyl glucosyl)quinic acid, which has found previously in *C. monogyna* leaves [2]. Peak 5 at 5.42 min at *m*/*z* 401.1449 with a fragment ion at *m*/*z* 269.1012 corresponds with benzyl alcohol-hexose-pentose, which was previously detected in *C. monogyna* leaves [2]. At 5.76 min (peak 6) at *m*/*z* 353.0873 with a molecular formula of C_16_H_17_O_9_ presented fragment ions at *m*/*z* 191.0550, 179.0339, 173.0447, 161.0231 and 135.0439; it was identified as chlorogenic acid (3-*O*-caffeoylquinic acid), which has been found in hawthorn leaves (*Crataegus grayana* Eggl., syn. *Crataegus flabellata* (Bosc ex Spach) K.Koch) leaves and in acerola fruit (*Malpighia punicifolia*, L., syn. *Malpighia glabra* L.) and *Crataegus monogyna* leaves [2,8,29]. Peak 7 at 6.15 min presented a molecular ion at *m*/*z* 371.0981 with molecular formula C_16_H_19_O_10_ and was proposed to be hydroferulic acid glucuronide, which has been found previously in tomatoes [30]. Peak 8 at 7.25 min and a molecular ion at *m*/*z* 337.0913 with fragments ion at *m*/*z* 191.0553, 163.0390 and 119.0492 was identified as cis-3-*O*-p-coumaroylquinic acid (C_23_H_13_O_3_), which was previously identified in *C. monogyna* leaves [2]. Peak 9 at 7.91 min with a molecular ion at *m*/*z* 433.1135 with fragment ions at *m*/*z* 313.0688 and 343.0743 was identified as naringenin C-hexoside (C_21_H_21_O_10_), and this was previously found in *C. monogyna* leaves [2]. Peak 10 at 9.09 min with a molecular ion at *m*/*z* 577.1561 and fragment ion at *m*/*z* 293.0446, 413.0870, 311.0551 and 457.1120 was proposed as 2″-*O*-rhamnosyl-*C*-hexosyl-apigenin (C_27_H_29_O_14_), which was previously identified in *C. monogyna* flower buds [9]. Peaks 11 and 12 at 10.13 and 10.32 min with a molecular ion at *m*/*z* 609.1456 with fragments ions at *m*/*z* 301, 300 and 271 were identified as isomers of quercetin 3-*O*-rutinoside (C_27_H_29_O_16_), which were already identified in *C. monogyna* leaves [2]. Peak 13 at 10.39 min with a molecular ion at *m*/*z* 609.1479 and fragment ions at *m*/*z* 301.0335 and 300.0274 was proposed to be quercetin-3-*O*-galactoside (hyperoside) (C_21_H_19_O_12_) previously reported in *C. monogyna* leaves [2]. Peak 14 at 10.53 min with a molecular ion at *m*/*z* 463.0881, presenting a molecular formula of C_21_H_19_O_12_ and fragment ion at *m*/*z* 301.035, corresponds with quercetin-3-*O*-glucoside (isoquercetin) [2]. Kaempferol-3-O-glucoside (astragalin) was detected at 11.11 min (peak 15) with a molecular ion at *m*/*z* 447.0918 with fragment ions at *m*/*z* 285.0378, 255.0285 and 284.0304 and a molecular formula of C_21_H_19_O_11_, which was previously detected in *C. monogyna* leaves [2]. Peak 16 at 11.22 min with a molecular ion at *m*/*z* 433.076 and a fragment ion at *m*/*z* 301.031 with a molecular formula of C_20_H_17_O_11_ corresponds with quercetin-pentoside, which has been detected previously in *C. monogyna* fruit extracts and in *Crataegus grayana* fruits and leaves [9]. At retention time 11.43 min (peak 17) and *m*/*z* 505.0973, peak 17 was identified as quercetin-*O*-acetyl hexoside with a molecular formula of C_23_H_21_O_13_ with fragment ions at *m*/*z* 463.0902 and 301.0323 [2]. Peak 18 at 11.7 min with a molecular ion at *m*/*z* 451.1028 with fragment ions at *m*/*z* 341.0648, 289.0706 and 217.0145 was detected as cinchonain Ia and it has been identified in *C. monogyna* fruit [2]. Peak 19 at 12.6 min with a molecular ion at *m*/*z* 461.239 was tentatively identified as methyl luteolin-C-hexoside, which has been identified in *Crataegus grayana* fruits and leaves [8].

### 2.5. Identification and Quantification of Proanthocyanididins in C. monogyna Optimum Leaf Extract by NP-HPLC-FLD-MS

A total of 11 proanthocyanidins were identified in *C. monogyna* leaf extract according to their degree of polymerization and their mass spectra.

The elution order depended on the number of flavan-3-ol units. Therefore, monomers eluted first and then the oligomers eluted [31]. The identification of proanthocyanidins was confirmed by HPLC-ESI-TOF-MS.

### 2.6. Quantification of Phenolic Compounds in C. monogyna Optimum Leaf Extract by HPLC-MS

Optimum *C. monogyna* leaf extract was analyzed by HPLC-MS. A total of 19 phenolic compounds were quantified: seven phenolic acid derivatives, seven flavonols, two flavones, one flavonolignan, one flavanone, and one simple phenol. The calibration curves were used to quantify the phenolic compounds in *C. monogyna* optimum extract. All calibration curves showed a good linearity (*r^2^* > 0.9954). The limit of detection (LOD) and limit of quantification (LOQ) ranged between 0.04 and 0.47 mg/L, and 0.14 and 1.57 mg/L.

Table 5 shows the quantitative results of these phenolic compounds in *C. monogyna* leaf extract. Phenolic acid derivatives were the most concentrated phenolic compounds in *C. monogyna* optimum extract. Among phenolic acid derivatives, the most concentrated were dihydroxy benzoic acid pentoside, followed by protocatechuic acid glucoside and chlorogenic acid, which represent 28.2%, 26.9% and 23.2% of total phenolic acid derivatives. In addition, the most concentrated flavone was 2″-*O*-rhamnosyl-*C*-hexosyl-apigenin, which represents 99% of total flavones. Furthermore, the most concentrated flavonols were quercetin-3-galactoside (hyperoside) and quercetin-3-*O*-glucoside (isoquercetin), which represent 50.2% and 34.2% of the sum of flavonols. By comparison of these results with a previous study on *C. monogyna* extract, the content of chlorogenic acid and rutin was in the same magnitude order as that reported in two *C. monogyna* extracts (5.39 and 17.69 mg/g d.w. of chlorogenic acid and 0.36 and 1.28 mg/g dw. of rutin), whereas the content of hyperoside and isoquercetin were 45.6 and 81.9% higher than the mean reported by this previous study (2.43 and 0.55 mg/g d.w.) [25].

### 2.7. Quantification of Proanthocyanidins in C. monogyna Optimum Leaf Extract by HPLC-FLD

The quantification of proanthocyanins in *C. monogyna* leaf extract was carried out using HPLC-FLD. The calibration curve of the standard catechin was used to quantify the proanthocyanins (y = 88.157x + 143.99, r^2^ = 0.999). The correction factors were applied according with those established by Robbins et al. 2009 [32]. Table 6 reported the proanthocyanidins content and the HPLC-MS data of identification.

The concentration values of proanthocyanidins obtained in *Crataegus monogyna* leaf extract at optimum bath conditions appear in the Table 6. The most concentrated proanthocyanidin was catechin/epicatechin, where the value was similar to that reported by a previous study on hawthorn (*C. orientalis* Pall. ex M.Bieb) leaves (7.2 ± 0.04 mg/g d.w.) [24]. In addition, the total content of proanthocyanidins in *C. monogyna* leaf extract obtained at optimum UAE conditions was in concordance with a previous study that reported that procyanidin distribution during the seasonal growth of fresh plants of *Crataegus monogyna* showed a range of between 20 and 55 mg/g d.w. of procyanidins during the growing season in the different plant organs [17].

## 3. Materials and Methods

### 3.1. Samples

Sampling was done in the province of Granada (Spain). Briefly, 3 kg of *Crataegus monogyna* leaves were collected in the Huétor Santillán municipality (37°13′07″ N 3°31′02″ O) at about 1200 m of elevation. A voucher was included in the University of Granada Herbarium (GDA, Leg. G. Benítez). The fresh leaves with a moisture of 71.1 ± 6.5% were air dried at room temperature in a dark chamber and they were then pulverized using an A 10 basic miller from IKA (IKA, Staufen, Germany). The milled leaves were sieved obtaining an average particle size of 0.2 mm.

### 3.2. Chemicals

HPLC-grade acetonitrile and acetone were purchased from Merck KGaA (Darmstadt, Germany) and water was purified using a Milli-Q system (Millipore, Bedford, MA, USA). Vanillic acid, chlorogenic acid, ferulic acid, quercetin, catechin and rutin were purchased from Sigma-Aldrich (St. Louis, MO, USA).

### 3.3. Experimental Design

A Box–Behnken design with 3 variables was carried out in order to optimize the extraction parameters to obtain the highest content of phenolic compounds in *C. monogyna* leaves. In this study, the three independent variables were acetone/water (X_1_), time (X_2_) and ratio (X_3_), with 3 levels for each variable and the response variables (Y) were the total phenolic content (TPC) obtained by Folin–Ciocalteu and the antioxidant capacity obtained by FRAP, DPPH and ABTS (Table 1). The percentage of acetone/water was 20, 50 and 80% (*v*/*v*), which was chosen based on previous studies that reported 70% of acetone/water (*v*/*v*) for the recovery of proanthocyanidins in hawthorn fruit stone and leaves [17,33]. The extraction time (10, 50 and 90 min) was similar to a previous study concerning the extraction of phenolic compounds from *Psidium guajava* L. leaves (5–55 min) [20]. In addition, the solvent-to-solid ratio was 20, 1000 and 1980 (*v*/*w*), where the lower value was chosen based on a previous study on hawthorn fruit stones [33]. Experiments were randomized to maximize the effects of unexplained variability in the observed responses, due to extraneous factors. The design consisted of 15 combinations including three center points (Table 1).

Response surface methodology (RSM) is the most popular tool for modeling. In RSM, a second-order polynomial equation below is always employed to build the relationship between the response variables and independent variables [34]. The experimental design and the determination of optimal UAE bath parameters of an experiment based on the higher concentration of total phenolic compounds were carried out using STATISTICA 7.0 (2002, StatSoft, Tulsa, OK, USA).

### 3.4. Extraction of Phenolic Compounds in C. monogyna Leaves Ultrasonic-Assisted Extraction

The extractions were carried out by adding the quantity of *C. monogyna* leaves with 10 mL of the selected solvent. The extraction was achieved by using an ultrasonic bath (Bandelin, Sonorex, RK52, Berlin, Germany) operating at a frequency of 35 kHz. The percentage of acetone/water, extraction time and the US power were varied according to the experimental design. After the extraction, centrifugation was carried out at 1000 g for 10 min, the supernatant was then collected, evaporated and reconstituted in 1 mL of methanol/water (1:1, *v*/*v*). The final extracts were filtered through 0.2 μm nylon syringe filters and stored at −18 °C until the analyses.

### 3.5. Determination of Total Phenolic Content by Folin–Ciocalteu

Total phenolic compounds were determined by the Folin–Ciocalteu spectrophotometric method [35]. For that purpose, 100 µL of the extract was added to 500 µL of Folin–Ciocalteu reagent and 6 mL of MilliQ water. The flask was agitated for a minute. After that, 2 mL of 15% (*w*/*v*) Na_2_CO_3_ was added and made up to 10 mL with MilliQ water and stored at dark conditions. The measurements were carried out after 2 h at 750 nm and 25 ◦C with a UV-visible spectrophotometer (Spectrophotometer 300 Array, UV–Vis, single beam, Shimadzu, Duisburg, Germany). A calibration curve obtained by standards of gallic acid (1, 5, 10, 25, 50, 100 and 250 ppm) was used to quantify the total phenolic content (TPC). TPC is expressed as mg gallic acid equivalents (GAE)/g dry weight (d.w.).

### 3.6. Antioxidant Capacity

Three different assays were used to determine the antioxidant capacity of the extracts obtained from olive leaf cultivars. In all assays, trolox (6-hydroxy-2,5,7,8-tetramethylchromen-2-carboxylic acid) was used as the standard and the results were expressed in mg of trolox equivalents (TE)/g of dry weight.

#### 3.6.1. DPPH Radical Scavenging

A DPPH assay was performed according to the procedure described previously [36]. The absorbance at 517 nm at 25 °C after 30 min was measured when 0.1 mL of the samples were added to 2.9 mL of 100µM DPPH (2,2-diphenyl-1-picrylhydrazyl) methanol/H_2_O 4/1 (*v*/*v*) solution.

#### 3.6.2. ABTS Cation Radical Scavenging

This assay was carried out following the method described by Re R. et al., 1999 [37]. ABTS radical cation (ABTS^+^) was generated by reacting 7 mM ABTS (2,2′-azino-di(3-ethylbenzothiazoline-6-sulfonic acid) stock solution with 2.45 mM potassium persulfate. This solution was incubated for 12 h at room temperature and protected from light. Then, the ABTS reagent was diluted with EtOH until reaching an absorbance of 0.7 ± 0.02 at 734 nm at 30 °C. The assay consisted in the addition of 10 µL of extracts to 1mL of diluted ABTS reagent and measuring the decrease in absorbance after 10 min.

#### 3.6.3. Ferric Reducing Antioxidant Power (FRAP)

A FRAP assay was done according to a method previously described [38]. Briefly, 30 µL of the extracts was diluted with 90 µL of water, which was added to 0.9 mL of FRAP reagent. The FRAP reagent was prepared by mixing 25 mL of 0.3 mM acetate buffer (pH 3.6); 2.5 mL of 10 mM of TPTZ (2,4,6-Tri(2-pyridyl)-1,3,5-triazine) in 40 mM HCl solution and 2.5 mL of 20 mM FeCl3·6H2O solution. The absorbance was measured at 595 nm after 30 min.

### 3.7. Determination of Phenolic Compounds in Crataegus monogyna Extracs by HPLC-ESI-TOF-MS Analysis

*C. monogyna* leaf extract obtained at optimum ultrasonic-assisted extraction conditions was analyzed by an ACQUITY Ultra Performance LC system (Waters Corporation, Milford, MA, USA) coupled to an electrospray ionization (ESI) source operating in the negative mode and a time-of-flight (TOF) mass detector (Waters Corporation, Milford, MA, USA). Phenolic compounds were separated on an ACQUITY UPLC BEH Shield RP18 column (1.7 µm, 2.1 mm × 100 mm; Waters Corporation, Milford, MA, USA) at 40 °C using a gradient previously stated by Verni et al. 2020 [39] using water containing 1% acetic acid as mobile phase A and acetonitrile as mobile phase B. The data were elaborated using MassLynx 4.1 software (Waters Corporation, Milford, MA, USA).

### 3.8. Determination of Procyanidins in Crataegus Leaf Extract by HPLC-FLD

The separation of procyanidins was performed on an Agilent 1200 Series HPLC system) (Agilent Technologies, Santa Clara, CA, USA) equipped with a binary pump delivery system, a degasser, an autosampler, and a FLD. The column used was the Develosil Diol 100 Å (250 × 4.6 mm, 5 µm particle size) purchased from Phenomenex (Torrance, CA, USA). All solvents were HPLC grade and were filtered in a filter disk of 0.45 μm. The gradient elution that was carried out was the same reported by Robbins et al. 2009 [32]. Fluorescence detection was conducted with an excitation wavelength of 230 nm and an emission wavelength of 321 nm. The injection volume was 5 μL. All the analyses were carried out at a temperature of 35 °C. Calibration curves of (+)-catechin were arranged from 10 to 1330 μg/mL, respectively, and the correction factors suggested by Robbins et al. 2009 [32] were used for the quantification of dimers, trimers, tetramers, pentamers and the polymer (see Supplementary Materials).

## 4. Conclusions

In conclusion, a Box–Behnken experimental design was used in order to optimize the ultrasound-assisted extraction parameters to obtain the maximum phenolic content and antioxidant activities from *C. monogyna* leaves. The highest value of phenolic content and DPPH, ABTS and FRAP was obtained at 50% acetone/water (*v*/*v*), 55 min, and 1/1000 of solvent-to-solid ratio (*v*/*w*). Phenolic compounds in *Crataegus monogyna* leaf extract obtained at these optimum UAE conditions were analyzed by advanced analytical platforms; HPLC-ESI-TOF-MS permits the identification and quantification of 19 phenolic compounds (excluding flavan-3-ols) and, to our knowledge, most of them are identified for the first time in hawthorn leaves. HPLC-FLD-MS analyses showed the presence of 11 proanthocyanidins at different degree of polymerization. According to the results, *C. monogyna* leaf extract is a rich source of phenolic acid derivatives such as protocatechuic acid-glucoside, dihydroxy benzoic acid pentoside and chlorogenic acids, flavones such as 2″-*O*-rhamnosyl-*C*-hexosyl-apigenin and flavonols such as hyperoside and isoquercetin. In addition, the most concentrated proanthocianidins were the monomer and dimer, which represent 49.0% and 21.1% of the total proanthocianidin content. Therefore, the optimum UAE conditions could be applied for the screening of phenolic compounds in *C. monogyna* leaves. Indeed, *C. monogyna* may be considered a rich source of phenolic compounds (flavonols, flavones, phenolic acids and proanthocyanidins) with bioactive activities to be used for pharmaceutical applications.

## Figures and Tables

**Table 1:** Experimental Box–Behnken design (BBD), with natural and coded values for the factors, and response variable values.

Run	Independent Factors			Dependent Factors			
	X_1_	X_2_	X_3_	TPC	DPPH	ABTS	FRAP
1	20	10	1000	56.73	60.31	72.59	65.95
2	80	10	1000	67.84	64.22	69.26	79.15
3	20	90	1000	64.24	37.68	82.21	109.22
4	80	90	1000	67.11	75.2	45.71	87.51
5	20	50	20	62.12	54.92	54.87	77.07
6	80	50	20	57.29	41.05	37.61	54.58
7	20	50	1980	53.14	75.05	62.06	82.23
8	80	50	1980	69.31	73.4	70.89	91.89
9	50	10	20	50.3	40.53	40.41	49.62
10	50	90	20	68.27	69.74	70.45	90.46
11	50	10	1980	38.04	54.03	86.7	65.54
12	50	90	1980	66.48	85.11	90.67	117.16
13	50	50	1000	78.6	93.04	98.18	134.68
14	50	50	1000	75.44	99.44	104.3	134.02
15	50	50	1000	78.32	101.3	102.43	125.51

X_1_: acetone/water ratio (% (*v*/*v*)); X_2_: time (min); and X_3_: solvent-to-solid ratio (*v*/*w*). TPC was expressed as mg gallic acid eq/g. d.w. DPPH, ABTS and FRAP were expressed as mg trolox/g sample d.w.

**Table 2:** Regression coefficients and analysis of variance (ANOVA) of the model for the response variables.

	TPC	DPPH	ABTS	FRAP
β_0_	33.78210 *	−4.23490	−24.5001 *	−31.9747
Linear				
β_1_	0.49921 *	1.86645 *	2.6944 *	2.7900 *
β_2_	0.75200 *	0.95654 *	1.1523 *	2.0480 *
β_3_	0.01257 *	0.03980 *	0.0532 *	0.0559 *
Cross product				
β_12_	−0.00170	0.00700 **	−0.0069 *	−0.0073 **
β_13_	0.00018 *	0.00010	0.0002 **	0.0003 **
β_23_	0.00007	0.00001	−0.0002 **	0.0001
Quadratic				
β_11_	−0.00487 *	−0.02212 *	−0.0277 *	−0.0279 *
β_22_	−0.00567 *	−0.01167 *	−0.0058 *	−0.0130 *
β_33_	−0.00001 *	−0.00002 *	−0.0000 *	−0.0000 *
R^2^	0.85971	0.80642	0.92192	0.97543
R^2^_adujsted_	0.84991	0.80004	0.91427	0.97271
CV	0.81067	0.56165	0.58919	0.19745
*p* (Lack of fit)	0.052959	0.076608	0.056799	0.326531

* Significant at *p* < 0.05 level, ** Significant at *p* < 0.01 level.

**Table 3:** Optimal conditions for UAE extraction.

Optimal Conditions	TPC	DPPH	ABTS	FRAP
Acetone/water % (*v*/*v*)	50	50	50	50
Time (min)	55	55	55	55
Solvent-to-solid ratio (*v*/*w*)	1000	1000	1000	1000
Predicted	78 ± 4	98 ± 11	102 ± 8	133 ± 13
Observed	78.9 ± 0.4	101 ± 2	103 ± 2	135 ± 2
Significant differences	N.S.	N.S.	N.S.	N.S.

N.S.: no significant differences. TPC was expressed as mg gallic acid eq/g d.w. DPPH, ABTS and FRAP were expressed as mg trolox/g sample d.w.

**Table 4:** Table of identification of phenolic compounds from optimum *C. monogyna* leaf extract by HPLC-MS.

Peak	RT	*m*/*z* Experimental	*m*/*z* Calculated	Tolerance (ppm)	Error (ppm)	Fit Conf %	In Source Fragments	Molecular Formula	Compound
1	1.93	315.0719	315.0716	20	1	99.02	153.0171, 109.0246	C_13_H_15_O_9_	Protocatechuic acid-glucoside
2	2.41	299.0759	299.0767	20	−2.7	61.83	137.0249	C_13_H_15_O_8_	Hydroxybenzoylhexose
3	3.63	285.0606	285.061	20	−1.4	99.99	152.0104, 153.0187, 108.0197, 109.0269	C_12_H_13_O_8_	Dihydroxy benzoic acid pentoside
4	5.32	515.1407	515.1401	20	1.2	69.65	191.0552, 161.0229, 323.0756	C_22_H_27_O_14_	5-*O*-(3′-*O*-Caffeoyl glucosyl) quinic acid
5	5.42	401.1449	401.1448	20	0.2	97.62	269.1012	C_18_H_25_O_10_	Benzyl alcohol- hexose-pentose
6	5.76	353.0873	353.0872	20	−0.3	100	191.0550, 179.0339, 173.0447, 161.0231, 135.0439	C_16_H_17_O_9_	Chlorogenic acid
7	6.15	371.0981	371.0978	20	0.8	99.59		C_16_H_19_O_10_	Hydroferulic acid glucuronide
8	7.25	337.0913	337.0865	20	4.2	96.47	191.0553, 163.0390, 119.0492	C_23_H_13_O_3_	*Cis*-3-*O*-*p*-coumaroylquinic acid
9	7.91	433.1132	433.1135	20	0	94.42	313.0688, 343.0743	C_21_H_21_O_10_	Naringenin *C*-hexoside
10	9.09	577.1561	577.1557	20	0.7	99.66	293.0446, 413.0870, 311.0551, 457.1120	C_27_H_29_O_14_	2″-*O*-rhamnosyl-*C*-hexosyl-apigenin
11	10.13	609.1456	609.1456	20	0	93.46	301.0325, 300.0263, 271.0242	C_27_H_29_O_16_	Quercetin 3-*O*-rutinoside (rutoside) isomer a
12	10.32	609.1479	609.1456	20	3.8	85.12	301.0331, 300.0272, 271.0242	C_27_H_29_O_16_	Quercetin 3-*O*-rutinoside (rutoside) isomer b
13	10.39	463.0879	463.0877	20	0.4	99.95	301.0335, 300.0274	C_21_H_19_O_12_	Quercetin-3-*O*-galactoside (Hyperoside)
14	10.53	463.0881	463.0877	20	0.9	99.79	301.035	C_21_H_19_O_12_	Quercetin-3-*O*-glucoside (Isoquercetin)
15	11.106	447.0918	447.0927	20	−2	91.01	285.0378, 255.0285, 284.0304	C_21_H_19_O_11_	Kaempferol -3-*O*-glucoside (astragalin)
16	11.22	433.076	433.0771	20	−2.5	94.31	301.031	C_20_H_17_O_11_	Quercetin-pentoside
17	11.43	505.0973	505.0982	20	−1.8	99.42	463.0902, 301.0323	C_23_H_21_O_13_	Quercetin-*O*-acetyl hexoside
18	11.7	451.1028	451.1029	20	−0.2	98.85	341.0648, 289.0706, 217.0145	C_24_H_19_O_9_	Cinchonain Ia
19	12.6	461.239	461.2387	20	0.7	87.27		C_22_H_37_O_10_	Methyl luteolin-*C*-hexoside

**Table 5:** Table of quantification of phenolic compounds from *C. monogyna* optimum leaf extract by HPLC-MS expressed as mg∙g^−1^ d.w.

Phenolic Compounds	Concentration (mg/g d.w.)
Protocatechuic acid-glucoside	7.5 ± 0.1
Hydroxybenzoylhexose	1.16 ± 0.03
Dihydroxy benzoic acid pentoside	7.94 ± 0.05
5-*O*-(3′-*O*-caffeoyl glucosyl)quinic acid	1.28 ± 0.07
Benzyl alcohol- hexose-pentose	1.10 ± 0.04
Chlorogenic acid (3-*O*-caffeoylquinic acid)	6.51 ± 0.09
Hydroferulic acid (HFA) glucuronide	2.79 ± 0.02
*Cis*-3-*O*-*p*-coumaroylquinic acid	0.855 ± 0.006
Naringenin *C*-hexoside	0.198 ± 0.001
2″-*O*-rhamnosyl-*C*-hexosyl-apigenin	8.911 ± 0.008
Quercetin 3-*O*-rutinoside (rutoside) isomer a	0.94 ± 0.02
Quercetin 3-*O*-rutinoside (rutoside) isomer b	0.37 ± 0.01
Quercetin-3-galactoside (hyperoside)	4.47 ± 0.04
Quercetin-3-*O*-glucoside (isoquercetin)	3.041 ± 0.02
Kaempferol 3-*O*-glucoside (astragalin)	0.0471 ± 0.0002
Quercetin-pentoside	0.0033 ± 0.0001
Quercetin-*O*-acetyl hexoside	1.31 ± 0.01
Cinchonain Ia	0.017 ± 0.002
Methyl luteolin-*C*-hexoside	0.0966 ± 0.0009
Sum of Flavonols	8.9 ± 0.1
Sum of Flavones	9.007 ± 0.009
Sum of phenolic acid derivatives	28.1 ± 0.1
Sum of phenolic compounds	48.6 ± 0.3

**Table 6:** Identification and quantification of proanthocyanidins from *C. monogyna* optimum leaf extract by HPLC-ESI-TOF-MS and HPLC-FLD. The results are expressed as mg∙g^−1^ d.w.

Proanthocyanidins	(M−H)^−^	Concentration (mg/g d.w.)
Monomers	289	8.7 ± 0.2
dp2	577	3.7 ± 0.3
dp3	865	2.09 ± 0.02
dp4	1153	1.09 ± 0.08
dp5	1441	0.565 ± 0.03
dp6	-	0.258 ± 0.06
dp7	-	0.174 ± 0.007
dp8	-	0.0723 ± 0.0004
dp9	-	0.034 ± 0.001
dp10	-	0.0165 ± 0.0002
Polymers	-	0.76 ± 0.05
Total	-	17.5 ± 0.5

dp = degree of polymerization.

## Data Availability

Not applicable.

## Supplementary Materials

The following are available online. Table S1. Calibration curves of standards. Table S2. HPLC-ESI-MS data of proanthocyanidins in *C. monogyna* leaf extract obtained at optimum ultrasonic assisted extraction conditions.

## Sample Availability

Samples are available from the authors.

## References

[1] Ferioli F., Giambanelli E., D’Antuono L.F. (2020). Application of different analytical methods for the determination of phenolics and antioxidant activity in hawthorn (*Crataegus* spp.) bud and sprout herbal extracts. J. Appl. Bot. Food Qual..

[2] Elsadig Karar M.G., Kuhnert N. (2016). UPLC-ESI-Q-TOF-MS/MS Characterization of Phenolics from *Crataegus monogyna* and Crataegus laevigata (Hawthorn) Leaves, Fruits and their Herbal Derived Drops (Crataegutt Tropfen). J. Chem. Biol. Ther..

[3] Christensen K. (1992). Revision of Crataegus Sect. Crataegus and Nothosect. Crataeguineae (Rosaceae-Maloideae) in the Old World. Syst. Bot. Monogr..

[4] Guinda Á., Rada M., Delgado T., Gutiérrez-Adánez P., Castellano J.M. (2010). Pentacyclic Triterpenoids from Olive Fruit and Leaf. J. Agric. Food Chem..

[5] Muñoz-Garmendia F., Navarro C., Aedo C., Crataegus L., Castroviejo S. (1998). Plantas Vasculares de la Península Ibérica e Islas Baleares.

[6] Alirezalu A., Ahmadi N., Salehi P., Sonboli A., Alirezalu K., Khaneghah A.M., Barba F.J., Munekata P.E.S., Lorenzo J.M. (2020). Physicochemical characterization, antioxidant activity, and phenolic compounds of hawthorn (*Crataegus* spp.) fruits species for potential use in food applications. Foods.

[7] Tadić V.M., Dobrić S., Marković G.M., Dordević S.M., Arsić I.A., Menković N.R., Stević T. (2008). Anti-inflammatory, Gastroprotective, Free-Radical-Scavenging, and Antimicrobial Activities of Hawthorn Berries Ethanol Extract. J. Agric. Food Chem..

[8] Liu P., Kallio H., Yang B. (2011). Phenolic compounds in hawthorn (*Crataegus grayana*) fruits and leaves and changes during fruit ripening. J. Agric. Food Chem..

[9] Rodrigues S., Calhelha R.C., Barreira J.C.M., Dueñas M., Carvalho A.M., Abreu R.M.V., Santos-Buelga C., Ferreira I.C.F.R. (2012). *Crataegus monogyna* buds and fruits phenolic extracts: Growth inhibitory activity on human tumor cell lines and chemical characterization by HPLC-DAD-ESI/MS. Food Res. Int..

[10] Yang B., Liu P. (2012). Composition and health effects of phenolic compounds in hawthorn (*Crataegus* spp.) of different origins. J. Sci. Food Agric..

[11] Pavlovic J., Mitic S., Mitic M., Kocic G., Pavlovic A., Tosic S. (2019). Variation in the phenolic compounds profi le and antioxidant activity in different parts of hawthorn (Crataegus pentagyna Willd.) during harvest periods. Polish J. Food Nutr. Sci..

[12] Nabavi S.F., Habtemariam S., Ahmed T., Sureda A., Daglia M., Sobarzo-Sánchez E., Nabavi S.M. (2015). Polyphenolic composition of *Crataegus monogyna* jacq.: From chemistry to medical applications. Nutrients.

[13] Scheffer J.J.C. (1991). Plants as source of new drugs. Pharm. Weekbl..

[14] Caliskan O. (2015). Mediterranean Hawthorn Fruit (Crataegus) Species and Potential Usage. The Mediterranean Diet: An Evidence-Based Approach.

[15] (2004). European Directorate for the Quality of Medicines & HealthCare (EDQM).

[16] Ngoc P.C., Leclercq L., Rossi J.C., Desvignes I., Hertzog J., Fabiano-Tixier A.S., Chemat F., Schmitt-Kopplin P., Cottet H. (2019). Optimizing water-based extraction of bioactive principles of hawthorn: From experimental laboratory research to homemade preparations. Molecules.

[17] Hellenbrand N., Sendker J., Lechtenberg M., Petereit F., Hensel A. (2015). Isolation and quantification of oligomeric and polymeric procyanidins in leaves and flowers of Hawthorn (*Crataegus* spp.). Fitoterapia.

[18] Verardo V., Cevoli C., Pasini F., MaríaGómez-Caravaca A., Marconi E., Fabbri A., Caboni M.F. (2015). Analysis of oligomer proanthocyanidins in different barley genotypes using High-Performance Liquid Chromatography—Fluorescence Detection − Mass Spectrometry and Near-Infrared Methodologies. J. Agric. Food Chem..

[19] Díaz-de-Cerio E., Pasini F., Verardo V., Fernández-Gutiérrez A., Segura-Carretero A., Caboni M.F. (2017). *Psidium guajava* L. leaves as source of proanthocyanidins: Optimization of the extraction method by RSM and study of the degree of polymerization by NP-HPLC-FLD-ESI-MS. J. Pharm. Biomed. Anal..

[20] Díaz-de-cerio E., Tylewicz U., Verardo V., Fernández-Gutiérrez A., Segura-Carretero A., Romani S. (2017). Design of Sonotrode Ultrasound-Assisted Extraction of Phenolic Compounds from *Psidium guajava* L. Leaves. Food Anal. Methods.

[21] Martín-García B., Pasini F., Verardo V., Díaz-De-cerio E., Tylewicz U., Gómez-Caravaca A.M., Caboni M.F. (2019). Optimization of sonotrode ultrasonic-assisted extraction of proanthocyanidins from brewers’ spent grains. Antioxidants.

[22] Le Man H., Behera S.K., Park H.S. (2010). Optimization of operational parameters for ethanol production from korean food waste leachate. Int. J. Environ. Sci. Technol..

[23] Pan G., Yu G., Zhu C., Qiao J. (2012). Optimization of ultrasound-assisted extraction (UAE) of flavonoids compounds (FC) from hawthorn seed (HS). Ultrason. Sonochem..

[24] Šavikin K.P., Krstić-Milošević D.B., Menković N.R., Beara I.N., Mrkonjić Z.O., Pljevljakušić D.S. (2017). Crataegus orientalis leaves and berries: Phenolic profiles, antioxidant and anti-inflammatory activity. Nat. Prod. Commun..

[25] Alirezalu A., Salehi P., Ahmadi N., Sonboli A., Aceto S., Maleki H.H., Ayyari M. (2018). Flavonoids profile and antioxidant activity in flowers and leaves of hawthorn species (*Crataegus* spp.) from different regions of iran. Int. J. Food Prop..

[26] Żurek N., Karatsai O., Rędowicz M.J., Kapusta I.T. (2021). Polyphenolic Compounds of Crataegus Berry, Leaf, and Flower Extracts Affect Viability and Invasive Potential of Human Glioblastoma Cells. Molecules.

[27] Lin L.Z., Sun J., Chen P., Zhang R.W., Fan X.E., Li L.W., Harnly J.M. (2014). Profiling of glucosinolates and flavonoids in rorippa indica (Linn.) Hiern. (cruciferae) by UHPLC-PDA-ESI/HRMSn. J. Agric. Food Chem..

[28] Russo D., Kenny O., Smyth T.J., Milella L., Hossain M.B., Diop M.S., Rai D.K., Brunton N.P. (2013). Profiling of Phytochemicals in Tissues from Sclerocarya birrea by HPLC-MS and Their Link with Antioxidant Activity. ISRN Chromatogr..

[29] Dissanayake A.A., Zhang C.R., Gaber M.K.A., Nair M.G. (2017). Salicylic glycosides in Salix mucronata with antioxidant and antiinflammatory activities. Nat. Prod. Commun..

[30] Martínez-Huélamo M., Vallverdú-Queralt A., Di Lecce G., Valderas-Martínez P., Tulipani S., Jáuregui O., Escribano-Ferrer E., Estruch R., Illan M., Lamuela-Raventós R.M. (2016). Bioavailability of tomato polyphenols is enhanced by processing and fat addition: Evidence from a randomized feeding trial. Mol. Nutr. Food Res..

[31] Verardo V., Gómez-Caravaca A.M., Marconi E., Caboni M.F. (2011). Air classification of barley flours to produce phenolic enriched ingredients: Comparative study among MEKC-UV, RP-HPLC-DAD-MS and spectrophotometric determinations. LWT Food Sci. Technol..

[32] Robbins R.J., Leonczak J., Johnson J.C., Li J., Kwik-Uribe C., Prior R.L., Gu L. (2009). Method performance and multi-laboratory assessment of a normal phase high pressure liquid chromatography-fluorescence detection method for the quantitation of flavanols and procyanidins in cocoa and chocolate containing samples. J. Chromatogr. A.

[33] Chai W.M., Chen C.M., Gao Y.S., Feng H.L., Ding Y.M., Shi Y., Zhou H.T., Chen Q.X. (2014). Structural analysis of proanthocyanidins isolated from fruit stone of chinese hawthorn with potent antityrosinase and antioxidant activity. J. Agric. Food Chem..

[34] Tao Y., Sun D.-W. (2015). Critical Reviews in Food Science and Nutrition Enhancement of Food Processes by Ultrasound: A Review. Crit. Rev. Food Sci. Nutr..

[35] Singleton V.L., Orthofer R., Lamuela-Raventós R.M. (1999). Analysis of total phenols and other oxidation substrates and antioxidants by means of Folin-Ciocalteu reagent. Methods Enzymol..

[36] Brand-Williams W., Cuvelier M.E., Berset C. (1995). Use of a free radical method to evaluate antioxidant activity. Leb. Technol..

[37] Re R., Pellegrini N., Proteggente A., Pannala A., Yang M., Rice-Evans C. (1999). Antioxidant activity applying an improved ABTS radical cation decolorization assay. Free Radic. Biol. Med..

[38] Pulido R., Bravo L., Saura-Calixto F. (2000). Antioxidant activity of dietary polyphenols as determined by a modified ferric reducing/antioxidant power assay. J. Agric. Food Chem..

[39] Verni M., Pontonio E., Krona A., Jacob S., Pinto D., Rinaldi F., Verardo V., Díaz-de-Cerio E., Coda R., Rizzello C.G. (2020). Bioprocessing of Brewers’ Spent Grain Enhances Its Antioxidant Activity: Characterization of Phenolic Compounds and Bioactive Peptides. Front. Microbiol..

