# Supplementary material for: Setup of an Ultrasonic-Assisted Extraction to Obtain High Phenolic Recovery in Crataegus monogyna Leaves
Source: Molecules. 2021 Jul 27;26(15):4536. doi: 10.3390/molecules26154536 (PMC8347228; doi:10.3390/molecules26154536)
Supplement: Supplementary file 1 [file molecules-26-04536-s001.zip › molecules-1300570-supplementary.pdf]

**Table S1.** Calibration curves of standards

| Analyte | LOD (mg/L) | LOQ (mg/L) | Calibration ranges (mg/L) | Calibration curves (mg/L) | $R^2$ |
|---|---|---|---|---|---|
| Vanillic acid | 0.47 | 1.57 | LOQ-236.67 | y = 21.069x + 197.15 | 0.9979 |
| Chlorogenic acid | 0.17 | 0.56 | LOQ-246.67 | y = 58.665x - 289.54 | 0.9984 |
| Ferulic acid | 0.27 | 0.89 | LOQ-226.67 | y = 37.071x + 155.61 | 0.9983 |
| Quercetin | 0.06 | 0.21 | LOQ-226.67 | y = 154.26x + 1309.1 | 0.9988 |
| Rutin | 0.04 | 0.14 | LOQ-220 | y = 239.6x + 690.3 | 0.9954 |

LOD: Limit of detection, LOQ: Limit of quantification

**Table S2.** HPLC-ESI-MS data of proanthocyanidins in *C. monogyna* leaf extract obtained at optimum ultrasonic assisted extraction conditions.

| **Peak** | **Proanthocyanidins** | **$[M-H]^-$** |
|---|---|---|
| 1 | monomers | 289 |
| 2 | dimers | 577 |
| 3 | dp3 | 865 |
| 4 | dp4 | 1153 |
| 5 | dp5 | 1441 |
| 6 | dp6 | |
| 7 | dp7 | |
| 8 | dp8 | |
| 9 | dp9 | |
| 10 | dp10 | |
| 11 | polymers | |
